# Functional properties of bioactive compounds from *Spirulina* spp.: Current status and future trends

**DOI:** 10.1016/j.fochms.2022.100134

**Published:** 2022-09-19

**Authors:** Débora Gonçalves Bortolini, Giselle Maria Maciel, Isabela de Andrade Arruda Fernandes, Alessandra Cristina Pedro, Fernanda Thaís Vieira Rubio, Ivanise Guiherme Branco, Charles Windson Isidoro Haminiuk

**Affiliations:** a Universidade Federal do Paraná (UFPR), Programa de Pós-Graduação em Engenharia de Alimentos (PPGEAL), Curitiba, Paraná CEP (81531-980), Brazil; b Universidade Tecnológica Federal do Paraná (UTFPR), Departamento Acadêmico de Química e Biologia (DAQBi), Laboratório de Biotecnologia, Curitiba, Paraná CEP (81280-340), Brazil; c Universidade de São Paulo, Escola Politécnica, Department of Chemical Engineering, Main Campus, São Paulo, São Paulo 05508-080, Brazil; d Universidade Estadual Paulista (UNESP), Departamento de Ciências Biológicas, Assis, São Paulo, São Paulo 19806-900, Brazil

**Keywords:** Phycocyanin, Microalgae, Aminoacids, Unsaturated fatty acids, Phenolic compounds, Carotenoids

## Abstract

Functional foods show non-toxic bioactive compounds that offer health benefits beyond their nutritional value and beneficially modulate one or more target functions in the body. In recent decades, there has been an increase in the trend toward consuming foods rich in bioactive compounds, less industrialized, and with functional properties. Spirulina, a cyanobacterium considered blue microalgae, widely found in South America, stands out for its rich composition of bioactive compounds, as well as unsaturated fatty acids and essential amino acids, which contribute to basic human nutrition and can be used as a protein source for diets free from animal products. In addition, they have colored compounds, such as chlorophylls, carotenoids, phycocyanins, and phenolic compounds which can be used as corants and natural antioxidants. In this context, this review article presents the main biological activities of spirulina as an anticancer, neuroprotective, probiotic, anti-inflammatory, and immune system stimulating effect. Furthermore, an overview of the composition of spirulina, its potential for different applications in functional foods, and its emerging technologies are covered in this review.

## Highlights

• Functional foods that contain bioactive compounds (BC) and provide health benefits;
• Spirulina is a cyanobacterium considered blue microalgae rich in BC;
• BC from Spirulina have interesting health effects;
• Chlorophyll, carotenoids, and phycocyanin are natural corants from Spirulina;
• Spirulina has potential as an ingredient for application in functional foods.

## 1 Introduction

Currently, more and more consumers correlate eating habits and a healthy lifestyle to reduce the incidence of chronic diseases. In response to this awareness, food industries have been striving to reduce artificial additives while also developing products that provide essential nutrients and contain ingredients beneficial to health and improve physical and mental well-being (Bigliardi and Galati, 2013, Carpentieri et al., 2022). In this context, there is growing attention to research involving functional foods, and the development of this type of product has strongly influenced the market (Alongi & Anese, 2021).

In 2018, the value of the functional foods market was estimated at USD 161.49 billion, with a projection of USD 275.77 billion in 2025 Grand View Research, 2019a, Grand View Research, 2019b. Regarding publications, the attention to functional foods was also evident. In the last five years, 32,143 publications were found in the Web of Science database using “functional foods” as a keyword. The most common food products with functional claims on the market include yogurts (digestive health), cereals (heart health), margarines and butters (cholesterol metabolism), drinks, and energy or protein bars (appetite reduction) (Granato, Barba, Bursać Kovačević, Lorenzo, Cruz, & Putnik, 2020).

According to Gur, Mawuntu, and Martirosyan (2018), based on the definition proposed by the “Functional Food Center” (FFC), functional foods are natural or processed foods that contain adequate and non-toxic amounts of biologically active compounds and, therefore, provide health benefits, preventing or treating diseases or their symptoms. Alongi and Anese (2021) also add to the classification of functional foods which are fortified with ingredients that beneficially influence health; products whose anti-nutritional compounds are excluded; raw materials that are improved, fortified, or “cleaned” (e.g., low-sugar foods) by modifying agricultural practices or post-harvest treatments; and new products that provide improved health. The inclusion criteria for a food or ingredient to be considered functional involve food safety, free access without a prescription and evidence of health benefits when regularly consumed in a balanced diet (Granato et al., 2020).

The existence of several sources with functional properties to be investigated and explored still represents a challenge for the scientific community and the industrial sector (Lim, Chang, Fazry, Wan Mustapha, & Babji, 2021). Recent studies point to microalgae as emerging sources of functional ingredients (Grochowicz, Fabisiak, & Ekielski, 2022) since they represent a rich source of multiple macro and micronutrients, including proteins, carbohydrates, phenolic compounds, vitamins, and minerals. Furthermore, the amount and type of bioactive compounds present in food determine their effectiveness in different body functions. Therefore, a diet composed of products rich in some phytochemical components can reduce, for example, the risk of developing several chronic diseases, such as neurodegenerative diseases, diabetes, and cancer (Grochowicz et al., 2022).

Several studies have shown that incorporating algae and their isolated bioactive in the formulation of food products contributes to both technological and functional properties. The main technological properties englobe stabilizing and emulsifiers power (Rodrigues et al., 2020), modifications in color and flavor (Freitas et al., 2019), and increasing of shelf life (Carvalho, Moreira, Oliveira, & Costa, 2017). Whereas functional characteristics are correlated to increasing of protein, lipid, mineral and bioactive content (Almeida et al., 2021, da Silva et al., 2021b, Los et al., 2018, Lucas et al., 2020, Lucas et al., 2018, de Oliveira et al., 2021). However, few studies in the literature address in detail the important properties of Spirulina microalgae and their phytochemical compounds. In addition, several sources of Spirulina have not yet been explored and may have industrial importance. Thus, the knowledge of new sources and their properties allows the development of innovative products based on Spirulina microalgae and contributes to essential effects on human health.

**Fig. 1:**
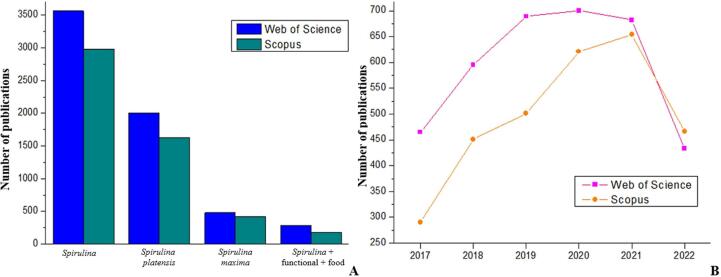
Number of publications found by searching for specific keywords (A) and over the last five years (B).

In this context, this review article brings together and addresses studies in different research areas in the last five years related to Spirulina genus microalgae of importance in South America. This review, which was elaborated on scientific articles published in the last five years, presents an overview of the composition and biological activity and the innovations and applications of Spirulina in the functional foods segment. The databases used were Science Direct, Google Scholar, and Web of Science, addressing 77 citations. The main keywords used in the searches were: functional properties, functional foods, South American algae, Spirulina, *in vitro* and *in vivo* biological activities, innovations, and technological applications. Fig. 1 shows the number of publications searching for specific keywords and periods. Papers were selected accordingly to their publication date (preferably less than five years). Articles without impact factor were not considered relevant for citation.

## 2 Composition of microalgae from Spirulina

**Fig. 2:**
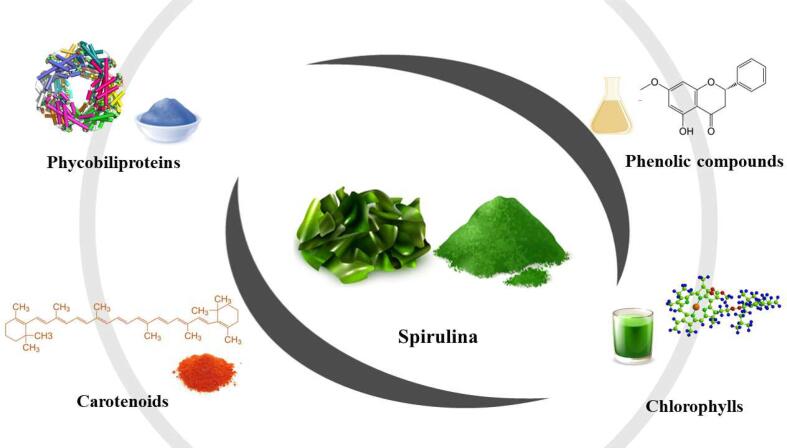
Bioactive compounds of Spirulina.

Microalgae, especially from the Spirulina genus, have an interesting nutritional composition, including macronutrients such as carbohydrates, lipids, proteins, and vitamins and minerals, which are essential for basic human nutrition (Table 1). Also, to macro and micronutrients, Spirulina has other compounds with biological activities. The main bioactive compounds in cyanobacteria, such as Spirulina, are characterized by unsaturated fatty acids, amino acids, carotenoids, and phenolic compounds (Fig. 2). These compounds are responsible for different biological activities such as antioxidant (Grover et al., 2021), anticarcinogenic (Tajvidi et al., 2021), and neuroprotective function (Haider et al., 2021).

**Table 1:** Centesimal composition of *Spirulina*.

Macronutrients(g/100 g FW)		Minerals (mg/g FW)		Vitamins (mg/g DW)		Fatty acids (mg/g FW)		Aminoacids (mg/g DW)		Bioactive compounds (g/100 g DW)	
Water	90.7 ^a^	Ca	1.05–12 ^a b^	Vit C	0.9 ^a*^	SFA 14:0	4 ^a^	Tryptophan	8.5 ^e^	Gallic acid	0.69–1.98^f^
Protein	5.92 ^a^	Fe	0.15–2.79 ^a b^	Thiamin	48 × 10^-3c^	SFA 16:0	67.49 – 184.86 ^a d^	Threonine	33.1 ^e^	Hydroxybenzoic acid	0.76–1.42^f^
Fat	0.39 ^a^	Mg	1.42–19 ^a b^	Riboflavin	39 × 10^-3c^	SFA 18:0	4 – 14.59 ^a d^	Isoleucine	36.4 ^e^	Chlorogenic acid	0.11–1.32^f^
Ash	0.6 ^a^	P	11 ^a^	Niacin	3.9^c^	SFA 11:0	5.42 – 16.22^d^	Leucine	61.7 ^e^	Vanillin	0.02–0.71^f^
Carbohydrate	2.42 ^a^	K	13.32–127 ^a b^	Pantothenic acid	0.325 ^a *^	MUFA 16:1	7.40–17 ^a d^	Lysine	34 ^e^	Caffeic acid	0.18–0.79^f^
Total dietary fiber	0.4 ^a^	Na	11.50–98 ^a b^	Vit B-6	90 × 10^-3c^	MUFA 18:1	18 – 248.01 ^a^	Methionine	17.1 ^e^	Syringic acid	0.08–0.52^f^
Total sugar	0.3 ^a^	Zn	(9.7 – 200) × 10^-3 a b^	Folic acid	7.3 × 10^-3c^	PUFA 18:2	16.66–64 ^a d^	Cystine	6.4 ^e^	Salicylic acid	0.02–0.41^f^
		Cu	0.579 ^a^	Choline	6.5 ^a*^	PUFA 18:3	42 – 307.35 ^ad^	Phenylalanine	33.3 ^e^	*O*-coumaric acid	0.01–0.41^f^
		Mn	(9.5 – 186) × 10^-3 a b^	Vit A	3 × 10^-3 a *^			Tyrosine	30.7 ^e^	Ferulic acid	0.30–0.72^f^
		Se	7 × 10^-3 a^	b-carotene	18 × 10^-3c^			Valine	42.2 ^e^	Cinnamic acid	0.11–1.81^f^
				Vit E	1.06^c^			Arginine	44.7 ^e^	Quercetin	0.11–0.63^f^
				Vit K	222 × 10^-3c^			Histidine	11.3 ^e^	Genstein	0–0.12^f^
								Alanine	50.2 ^e^	Euganol	0.11–0.73^f^
								Aspartic acid	63.1 ^e^	Galangin	0.05–0.33^f^
								Glutamic acid	84.7 ^e^	Pinostrobin	0.73–3.36^f^
								Glycine	34.3 ^e^	Phycocyanin	2.83 – 47.84 ^e g^
								Proline	25.3 ^e^	b-carotene	0.09–1.04 ^h^
								Serine	309 ^a*^	Canthaxanthin	0.44–0.65 ^h^
										Astaxanthin	0.1–0.72 ^h^
										Lutein	0.12–1.03 ^h^
										Zeaxanthin	0.03–0.61 ^h^
										Total chlorophylls	0.34 – 1.03 ^h^

Note: FW: Fresh weight. DW: Dry weight. Vit: Vitamin. SFA: Saturated fatty acid. MUFA: Monounsaturated fatty acid. PUFA: Polyunsaturated fatty acid. ^a^: USDA (2022) (*Spirulina* spp.). ^b^: Michael, Kyewalyanga, and Lugomela (2019) (*Arthrospira fusiformes*). ^c^: Masuda and Chitundu (2019) (*Spirulina platensis*). ^d^: de Morais et al. (2019) (*Spirulina* sp.). ^e^: Menegotto et al. (2019) (*Spirulina platensis*). ^f^: El-baky, El Baz, and El-baroty (2009) (*Spirulina máxima*). ^g^: Hynstova et al. (2018) (*Spirulina platensis*). ^h^: Rodrigues et al. (2018) (*Arthospira platensis*).* FW.

### 2.1 Macronutrients

The lipid profile is formed by saturated fatty acids (SFA), monounsaturated (MUFA), and polyunsaturated (PUFA) fatty acids (Table 1). Thus, Spirulina fatty acids, especially polyunsaturated fatty acids, have the potential to be used in specific diets for cases of lipid metabolism disorders (Li et al., 2019). Furthermore, Spirulina has the nine essential amino acids and other amino acids that together make up the proteins of this microalgae. This food matrix has all the essential amino acids, such as tryptophan, threonine, leucine, lysine, methionine, phenylalanine, histidine, and valine (Table 1) are not produced by the human body. So, Spirulina can be considered a source of essential and non-essential amino acids with the potential to enrich foods with low protein concentrations (Bashir, Sharif, Butt, & Shahid, 2016).

### 2.2 Micronutrients and bioactive composition

Phenolic compounds, characterized by molecules containing a benzene ring with at least one hydroxyl substituent, are the main examples of bioactive compounds found in products of plant origin, including teas (Bortolini, Haminiuk, Pedro, Fernandes, & Maciel, 2021), fruits, and their derivatives (Rossetto et al., 2020, Stafussa et al., 2021, Bortolini et al., 2022a), edible flowers (Bortolini et al., 2022b), and algae (Machu et al., 2015). Also, flavonoids and phenolic acids are the main classes of phenolic compounds reported in Spirulina (Table 1).

Other bioactive compounds such as chlorophyll, carotenoids, and phycocyanin contribute to the coloring of this type of algae. Carotenoids are fat-soluble compounds with colors varying between yellow and orange to reduce the damage caused by exposure to light in plants and microorganisms. The application of carotenoids in foods extends to additives with coloring and flavoring functions and vitamin A supplementation (Park et al., 2018). β-carotene, canthaxanthin, astaxanthin, lutein, and zeaxanthin are the main carotenoids found in Spirulina (Table 1).

Phycocyanin is a blue-colored water-soluble phycobiliprotein, stable at pH 5 – 8 (Park et al., 2018), found in blue algae (Table 1). Other important phycobiliproteins such as phycoerythrin (red) and allophycocyanin (blue) are found in microalgae from Spirulina genus (Rodrigues, de Castro, de Santiago-Aguiar, & Rocha, 2018). These bioactive substances can be used as dyes in the food industry, as there are few natural sources of blue colorants (Tavanandi & Raghavarao, 2020). The application of blue colorant can be mainly attractive for infant foods such as candies and some desserts, in which case phycocyanin can be a natural alternative to commonly used dyes (Faresin, Devos, Reinehr, & Colla, 2022).

Chlorophylls are other abundant bioactive compounds in Spirulina algae (Table 1). This phytochemical is responsible for this microalgae green color and has essential functions in the photosynthesis process (Martins et al., 2021). However, chlorophyll may be undesirable for foods because of its unattractive color for food application. In this context, some scientists have put some effort into masking this ingredient in the formulation of functional products.

As reported, Spirulina has an excellent nutritional and bioactive composition. Thus, the food and pharmaceutical industry can benefit from the biological activities found in the components of these matrices. Also, the presence of bioactive compounds allows for their use in different food formulations. Therefore, it can be emphasized for other groups, such as a protein supplement for vegans, blue dye for infant formulas, pro-vitamin A for the general people, and obtaining potentially functional foods.

## 3 *Spirulina* spp. production: Market and large-scale cultivation

*Spirulina* biomass has been certified as Generally Recognized as Safe (GRAS). Because of its long history of use (Lafarga, Fernández-Sevilla, González-López, & Acién-Fernández, 2020), it can be commonly found and commercialized in the market in the form of dry powder, capsules, and tablets (Thevarajah et al., 2022). Furthermore, *Spirulina* is one of the most worldwide cultivated microalgae (Silva, Ferreira, Dias, & Barreiro, 2020) and, according to a report published by Allied Market Research, the global *Spirulina* market achieved USD 393.6 million in 2019 (Allied Market Research, 2021), being expected to reach USD 968.6 million by 2028 (Meticulous Research, 2021). In terms of volume, the market is projected to reach 98,768.5 tons by 2028 (Meticulous Research, 2021). South America represents 8 % of the *Spirulina* powder market share, which is also segmented into North America (38 %), Europe (27 %), Asia-Pacific (21 %), and the rest of the world (6 %). Regarding the application, the food and beverages segment held the largest share in 2021 (Industry, 2021).

The current global market is dominated by artificially synthesized amino acids and blue pigments, which are constantly related to significant adverse effects on human health, such as severe allergic reactions, hypersensitive reactions and atrioventricular blocking capacity. Consequently, there is an increasing market demand for *Spirulina*-based proteins and c-phycocyanin to enhance the functional properties of food, pharmaceuticals, and nutraceuticals while maintaining health safety regulations. Although, there are several challenges associated with the cultivation and processing of Spirulina biomass, mainly the significant cost intensiveness during large-scale cultivation (Thevarajah et al., 2022).

*Spirulina* spp. develops naturally in saline alkaline lakes, especially subtropical and tropical lakes characteristics of Central America, South America, Africa, and Asia. However, they can also be found in hot fountains, swamps, and freshwater (Alagawany et al., 2021, Andrade et al., 2019). The genus spirulina has approximately 58 known species, among which *platensis* and *maxima* have higher productivity in laboratory conditions and for large-scale specific production (Alagawany et al., 2021). In general, the production of *Spirulina* spp. is done in Zarrouk standard medium, which contains carbon source (18.8 g/L of sodium bicarbonate, NaHCO_3_), mineral salts (potassium sulfate - K_2_SO_4_, dipotassium phosphate - K_2_HPO_4_, sodium chloride - NaCl, calcium chloride - CaCl_2_, magnesium sulphate - MgSO_4_ and iron(II) sulphate - FeSO_4_) and nitrogen source (sodium nitrate, NaNO_3_) (Andrade et al., 2019). However, this medium is considered expensive for producing *Spirulina spp* in high quantity, as it requires replacement by low-cost nutrients such as food co-products, commercial fertilizers, salt water, wastewater, and biomass reuse (Lafarga, Sánchez-Zurano, Villaró, Morillas-España, & Acién, 2021).

For large-scale production, the cultivation techniques of this microalgae depend on geographic location, temperature (30–35 °C), pH (9–11), light intensity (276–690 µmol m^−2^ s^−1^), cultivation time (30–40 days), strain type, inoculum quantity (10 % v/v), culture system (continuous, semi-continuous or batch), mixing and aeration (5–60 cm s^−1^), carbon source (dissolved NaHCO_3_, Na_2_CO_3_ or CO_2_) and carbon concentration (56.70–141.75 g h^−1^), nitrogen concentration, and producer knowledge (Iamtham and Sornchai, 2022, Thevarajah et al., 2022, Zhu et al., 2020).

Global production of *Spirulina* spp. reaches approximately 10,000 tons of dry biomass annually (Thevarajah et al., 2022), requiring high biomass productivity, determined by the optical density of cultures (Andrade et al., 2019). Among the cultivation parameters, the source in which inorganic carbon is obtained presents the most significant influence on the cultivation of *Spirulina spp* on a large scale since the microalgae require a large amount of this product to perform photosynthesis (Iamtham and Sornchai, 2022, Zhu et al., 2020). Due to this large amount, inorganic carbon is considered the most costly among the raw materials of the Zarrouk standard medium, which the carbon source can be cheapened by the use of wastewater rich in nitrate and phosphate, or organic compounds (commercial sugar, cassava flour, sweet potato flour, banana leaf ash extract), or bicarbonate salts and/or CO_2_-enriched air (Iamtham and Sornchai, 2022, Thevarajah et al., 2022).

Large-scale cultivation generally uses open pond systems (natural ponds, lakes, or raceway-type ponds) of different dimensions. However, it can be cultivated in closed or hybrid systems (Thevarajah et al., 2022). Zhu et al. (2020), for example, cultivated two *Spirulina* strains (*platensis* and *sp.*) in open-type raceway ponds with 605 m^2^ (length × width: 110×5.5 m and average depth of 32 cm) and with paddlewheel agitator (Power: 1.5 KW and rotation speed: 36 r min^−1^), presenting a dry biomass yield of 18.7 g m^−2^ d^−1^. The Earthrise Nutritionals LLC has 8.25 larger open ponds (5000 m^2^) and 37 farms and is therefore considered the largest producer of *Spirulina spp*. in the world (Thevarajah et al., 2022).

These open systems can be constructed of excavated concrete or polymer coatings (plastic alloy, vinyl, glass fibers) (Thevarajah et al., 2022, Zhu et al., 2020). Open systems have advantages such as ease of operation, simple design, low cost of operation, and investment. However, the risk of contamination is higher; it has lower biomass productivity compared to the closed system and difficulty controlling the cultivation parameters such as temperature and luminosity. On the other hand, although the closed system (column, tubular, or flat-plate photobioreactor) presents a difficult of operation and high cost of investment and capital, this type of system presents a lower risk of contamination, effective control of cultivation parameters, as well as higher productivity of biomass. Combining the two systems (integrated airlift systems, polybags, and external tubular loops) can maximize biomass and protein productivity. It facilitates the culture recirculation in the first stage cultivation and promotes an increase in the accumulation of photosynthetic pigment (c-phycocyanins) in two stages of cultivation (Thevarajah et al., 2022). Thus, meeting the demands of functional foods and emerging technologies.

## 4 *Spirulina*: Functional foods and emerging technologies

As previously stated, functional foods can have health benefits in addition to essential nutrition. Furthermore, health benefits must undergo randomized, double-blind, and placebo clinical trials inferring the product's functionality. This definition avoids doubts regarding using the terminology “functional food” since each country has only one regulation for classifying some foods as functional, but they do not have specific legislation (Granato et al., 2020). Therefore, the functionality of Spirulina-based foods should be carefully analyzed according to the criteria mentioned above.

Microalgae biomass can be an innovative source for the development of functional foods due to its natural origin and chemical composition and because it represents a rich source of bioactive compounds. Thus, microalgae consumption and their derivatives can positively affect consumer health (de Medeiros, da Costa, da Silva, Pimentel, & Magnani, 2021). In this context, Table 2 lists the works developed in South America in the last five years involving the application of Spirulina or extracts obtained from Spirulina in functional foods. Unfortunately, no studies were found applying other types of South American microalgae in functional foods.

**Table 2:** Recent studies showing the application of Spirulina in food products.

Application	Species	Main results	Reference
Electrolyte replenisher, a muscle enhancer, and recovery supplement	*Spirulina* sp. LEB-18	Three supplements focused on athletes were fortified with Spirulina. The enriched electrolyte replenisher had higher mineral content compared to a control formulation. The addition of Spirulina in the muscle enhancer led to an increase in carbohydrate content. No significant change was observed in the enriched recovery supplement compared to the control. Like existing commercial products, the developed food supplements had an estimated shelf life of between 9 and 11 months.	Carvalho et al. (2017)
Snacks	*Spirulina* sp. LEB-18	The extruded snacks produced with rice and corn flour and 2.6 % of free Spirulina had a higher content of proteins, minerals, and carotenoids than a control formulation without the microalgae. Although the snacks presented a green color, the presence of Spirulina biomass in the snacks did not negatively affect the product's sensory characteristics, and the acceptance rate was greater than 82 %. In addition, the food showed physical and microbiological stability over 12 months of storage.	Lucas et al. (2018)
Dried soup	*Spirulina platensis*	This study formulated dehydrated soups using peach palm by-products, Spirulina and spinach. The soup developed with Spirulina and peach palm by-product flour showed higher levels of proteins, lipids and antioxidants than the other formulations. In addition, compared to the soup formulated with peach palm and spinach by-products, it had a higher chlorophyll content. Regarding the low sensory acceptance, the authors highlight the need for further studies evaluating the amount of Spirulina that can be added to soups to improve the acceptance of formulations.	Los et al. (2018)
Protein concentrate	*Spirulina platensis*	Spirulina proteins were extracted and concentrated to produce a protein concentrate. The green–blue concentrate showed 75.97 % of proteins and 19.44 % of carbohydrates (in dry mass). All essential amino acids were found in the protein concentrate, indicating that the product is an alternative source of proteins to supply this nutrient. Furthermore, the foaming capacity of the concentrate presented percentages that compare to the egg yolk protein. In addition to the nutritional quality, the authors also highlight the functional importance of protein concentrate and its use to improve food processes.	Menegotto et al. (2019)
Shake to replace snacks and hypercaloric food	*Spirulina* sp. LEB-18	Comparing the products formulated with and without Spirulina, the unenriched formulations received higher scores in the sensory evaluation. The authors attributed this result to the effect of the characteristic color and flavor of the microalgae in the formulations. However, compared with similar commercial products, functional foods developed with Spirulina biomass had better sensory acceptance. In addition, more than 65 % of the panelists indicated purchase intention for the formulated products.	Freitas et al. (2019)
Pasta	*Spirulina* sp.	Spirulina microalgae was microencapsulated with sodium alginate and applied in pasta formulations. The formulations containing free and encapsulated Spirulina biomass presented an acceptability index greater than 70 % for the assessed attributes (color, texture, appearance, aroma, flavor, and overall liking), indicating that the incorporation of microalgae does not have a negative impact on the acceptability of the functional product. Although microencapsulation has protected the antioxidant activity of biomass and possibly has masked the perception of fish or algae odor in the formulated dough, the panelists did not perceive the presence of free Spirulina as a negative attribute in the product, which also encourages the development of products with the addition of free Spirulina.	Zen et al. (2020)
Snack bar	*Spirulina* sp. LEB-18	The authors formulated cereal bars applying 2 and 6 % of free Spirulina. With the increase in the addition of Spirulina, there was a significant increase in the protein concentration of the bars, and the green color became more pronounced. In the sensory evaluation, the panelists formed by children aged between 8 and 13 years, the appearance, flavor, and aroma attributes were well accepted. In addition to acting as a natural colorant in the production of bars, Spirulina biomass is also capable of nutritionally improving the foods in which it is applied and has shown to be a promising alternative in infant feeding.	Lucas et al. (2020)
Ice cream	*Spirulina* sp.	The authors demonstrated that it was possible to replace emulsifiers and stabilizing agents with the phycocyanin-rich extract from Spirulina. The extract showed emulsifying activity in oil-in-water (O/W) and in water-in-oil (W/O) emulsions. The addition of proteins present in the extract may have contributed to the development of texture in the product, influencing emulsification. The replacement of stabilizers and emulsifiers with phycocyanin extract did not change sensorially the overall acceptability of the product.	Rodrigues et al. (2020)
Ice cream	*Spirulina platensis* LEB-52	C-phycocyanin, a protein with bioactive properties, was extracted from Spirulina dry biomass, purified, and applied in an ice cream formulation. The product showed a blue color that remained stable over six months. The ice cream added with C-phycocyanin showed low antioxidant activity. However, after an *in vitro* digestion simulation, the product showed higher antioxidant activity than the control formulation. The authors highlight the C-phycocyanin extract as a stable colorant with biological action for application in food products and encourage studies that evaluate different colorant concentrations in the product's sensory characteristics.	de Amarante et al. (2020)
Chocolate milk	*Spirulina* sp. LEB-18	Spirulina biomass microencapsulated with maltodextrin and soy lecithin was incorporated into powdered chocolate milk formulations. The application of microalgae contributed to the increase in antioxidant activity, concentration of proteins and content of phenolic compounds in the formulations. In the sensory analysis, the average grades of the formulations, on a hedonic scale, indicated that the panelists neither liked nor disliked the product. Although the characteristic green color of the microalgae is not initially attractive in beverages, the formulated functional powder can be used in other food applications, such as cakes and cookies.	de Oliveira et al. (2021)
Biscuits	*Spirulina maxima* LEAF046	Free and encapsulated (using octenyl succinic anhydride starch) Spirulina biomass was added in biscuit formulations. A maximum of 10 % (w/w) application of free Spirulina was possible in the tested formulations. However, after the microencapsulation technique, 20 % (w/w) of Spirulina biomass was added without negative interference in the sensory quality of biscuits. Compared with the control biscuits, the samples enriched with free and encapsulated Spirulina demonstrated an average increase of 40 % in protein concentration.	da Silva, Valle, & Perrone (2021a)
Sauce	*Spirulina* sp.	The sauce formulated with the highest percentage of free Spirulina biomass (4 %) showed better scores for purchase intent and overall impression, being chosen as the best product evaluated. Compared to the control formulation, the sauce with 4 % of Spirulina showed higher concentrations of minerals, protein, fiber, monounsaturated fatty acids, and a significant increase in antioxidant activity. In addition, the ingredients used in the sauce formulations evaluated were able to mask the characteristic flavor of the microalgae.	Almeida et al. (2021)
Snacks	*Spirulina* sp. LEB-18	The authors developed extruded snacks by applying, in different formulations, non-hydrolyzed Spirulina, enzymatically hydrolyzed Spirulina and peptides isolated from Spirulina. The formulations added with isolated peptides showed higher antioxidant activity than the control sample. In addition, snacks added with peptides smaller than 4 kDa had a color closer to the control sample, which may be an interesting option for application in foods without negatively affecting the product's appearance.	da Silva et al. (2021b)
Ice cream	*Spirulina platensis*	Different ice cream formulations were produced by assessing the addition of inulin, Spirulina, and pigment extracted from Spirulina (phycocyanin) as emulsifiers and texturing agents, reducing the addition of sugars and fat. Regarding the texture of the ice creams, the formulations with the addition of inulin and phycocyanin-rich extract had better results. Although all formulations showed good acceptability, with scores between 6 (liked moderately) and 9 (liked extremely), the addition of Spirulina changed the color of the formulations and affected consumer acceptance. The addition of phycocyanin did not have the same effect.	Faresin, Devos, Reinehr, and Colla (2022)

Most of the Spirulina biomass produced today is consumed as a nutritional supplement promoted as a “superfood” and can be found in powder, flakes, or capsules (Lafarga et al., 2020). Also, Spirulina is recognized as safe for human consumption (Generally Recognized as Safe - GRAS). Its application in food has proved to be quite interesting from a nutritional point of view. Some authors report an increase in the protein concentration of the formulated product after the addition of Spirulina (Almeida et al., 2021, da Silva et al., 2021b, Los et al., 2018, Lucas et al., 2020, Lucas et al., 2018, de Oliveira et al., 2021). Moreover, by isolating and concentrating Spirulina proteins, Menegotto et al. (2019) identified all essential amino acids in the concentrate. The concentration of proteins from several microalgae species has been one of the main reasons to consider these organisms as an alternative source of protein. Microalgae represent a non-animal source of protein and have higher contents compared to beef, pork, and dairy products, also to superior amino acid quality (de Medeiros et al., 2021). Microalgae biomass can also contribute to adding bioactive compounds to the food, for example, carotenoids (Lucas, de Morais, Santos, & Costa, 2018), phenolic compounds (de Oliveira et al., 2021), and the blue pigment found in Spirulina, phycocyanin, which in addition to contributing to the color, is also characterized by its antioxidant potential (de Amarante et al., 2020, Faresin, Devos, Reinehr, & Colla, 2022; Rodrigues et al., 2020). With the addition of Spirulina in food formulations, authors also found an increase in the concentration of minerals (Almeida et al., 2021, Carvalho et al., 2017, Lucas et al., 2018).

According to the studies cited in Table 2, the most studied products were staple foods or foods with easy acceptance, such as cookies, pasta, sauce, ice cream, and snacks. Although functional foods have been shown to exert health benefits beyond the intrinsic effect of nourishing, it must be considered that the proposed formulations cannot be quite different from what consumers are used to eating, as consumer preferences are difficult to change. In this way, the approach involving foods with easy acceptance and more common foods can represent an opportunity to promote a healthier diet without requiring a change in consumer habits (Carpentieri et al., 2022). Extruded snacks have been studied increasingly due to the practicity of consumption and the variety of flavors, textures, and possible shapes. However, most formulations have low nutrients and may contain high sodium content (Lucas, de Morais, Santos, & Costa, 2017). Ice creams, in turn, are foods with high palatability. However, they have an increased range of fat and sugars (Faresin, Devos, Reinehr, & Colla, 2022). Therefore, both are potential options for studying alternative formulation changes. In addition, from the consumer's point of view, it is interesting to consume a sensorially superior product and, at the same time, to ingest compounds that are good for health.

It was also possible to verify the interest in developing products focused on energy replacement and for people who practice physical exercises (Carvalho et al., 2017, Freitas et al., 2019). Sports nutrition products were once consumed only by athletes or bodybuilders and almost untouchable by regular consumers. This scenario has changed with consumers' concomitant change and lifestyle, as they are increasingly informed about their nutritional choices and more health conscious. Not surprisingly, the industry has responded to this demand with a wide and growing range of powders, gels, drinks, and bars to enhance performance before, during, or after physical activity (Harrison & Smith, 2016). Spirulina consumption has shown results such as a protective effect against exercise-induced muscle damage, decreased blood lactate dehydrogenase levels, and lower serum glucose, cholesterol, and triglyceride levels (Freitas et al., 2019). Thus, Spirulina biomass is also an excellent choice for addition to products focused on sports nutrition.

Spirulina biomass has been applied in free and in encapsulated form. Encapsulation can play an essential role in masking algae's undesirable and characteristic odor or taste. Furthermore it can increase the stability of bioactive compounds, promote the delivery of active ingredients, prevent degradation due to external factors, improve solubility and facilitate the incorporation of bioactive compounds into systems or products (de Oliveira et al., 2021). In the study conducted by Zen et al. (2020), the authors report that microencapsulation was able to protect the antioxidant potential of Spirulina by 37.8 % during the cooking of the formulated pasta. Besides, the microparticles showed low water solubility; therefore, they are suitable for addition to products that require cooking in water.

On the other hand, de Oliveira et al. (2021), using maltodextrin and soy lecithin as carrier materials, observed an increase in the solubility of Spirulina microparticles compared to non-encapsulated biomass, which was advantageous for the formulation of chocolate milk powder. The authors also found a significant reduction in the average particle size after encapsulation and, therefore, easier application and less chance of being sensorially perceived. In the da Silva et al., 2021b study, microencapsulation allowed a more significant addition of Spirulina in the formulated vegan biscuits, most likely because the technique masked off-flavors from the biomass. However, the addition of Spirulina, encapsulated or not, had an evident effect on the color of the biscuits, affecting the appearance.

The contribution of chlorophylls to the color of microalgae is one of the most significant limitations of biomass application in products. It represents a challenge for the scientific community because the greenish tones can negatively affect the appearance of the evaluated product. In fact, color is responsible for a major impact on consumers sensory perception. Since it is the first characteristic seen, it may influence preference and purchase decisions (Gebhardt et al., 2020). However, the green color of Spirulina can represent an opportunity to innovate. Many food companies have been selling green foods and drinks in the last decade. In addition, the “marine” aroma and flavor of some microalgae can be an opportunity to develop new fish-based culinary preparations (Lafarga et al., 2020).

In recent work, Teixeira et al. (2022) mixed different concentrations of extracts of natural origin (hibiscus extract, water-soluble curcumin, and Spirulina extract). Then, they applied them to food models with different pH to compare the color obtained with the color provided by artificial colorants. As a result, the authors obtained mixtures with colors close to those of artificial dyes, indicating a potential alternative for replacing synthetic additives. In this case, Spirulina extract was not applied alone but as an adjuvant in the contribution of color. It is an option for the extract to add value to a product without the color being an issue.

In addition to chlorophyll, phycocyanin is a blue-colored protein found in Spirulina species. Its limitation is low stability at high temperatures and exposure to light. Therefore, its application has occurred exclusively in acidic foods that contain sugars or remain refrigerated, such as beverages, jellies, chewing gum, and ice cream (de Amarante et al., 2020, Neves et al., 2021). In the study carried out by de Amarante et al. (2020), the authors reported that ice cream formulated with C-phycocyanin extract showed stable color for the period studied of six months. The blue color is the biggest challenge among the natural colors, as natural blue sources are limited.

Furthermore, in nature, the blue color is rare compared to other colors; therefore, consumers often associate it with artificial ingredients (Neves et al., 2021). However, in the studies carried out by Rodrigues et al., 2020, Faresin, Devos, Reinehr, & Colla, 2022, the blue color of ice creams developed with phycocyanin had no negative influence on the sensory acceptance of the formulations. In both cases, the protein was not only responsible for the color but also presented functionality for the formulations, helping to develop texture. Thus, it is possible that the blue color is better accepted, even desired, in products that refer to children's taste, such as ice creams and candies, and may be a natural option for this type of product.

The potential of microalgae as a reliable source of proteins, minerals, and bioactive compounds for the development of functional foods is evident. Although the color of Spirulina and its derivatives and the taste and odor of the microalgae are still a limitation for its application, they represent an exciting challenge for future research, for example exploring techniques that mask the sensory characteristics and make the color of the product attractive, or that does not affect sensory acceptance - either through mixtures or addition to products in which the color in question is expected. Still, the overview of studies addressing the production of functional foods based on microalgae in South America clarifies the scarcity of studies. Furthermore, it highlights the need and opportunity to explore other types of microalgae for food application.

## 5 Biological effects of *Spirulina* spp. bioactive compounds

**Fig. 3:**
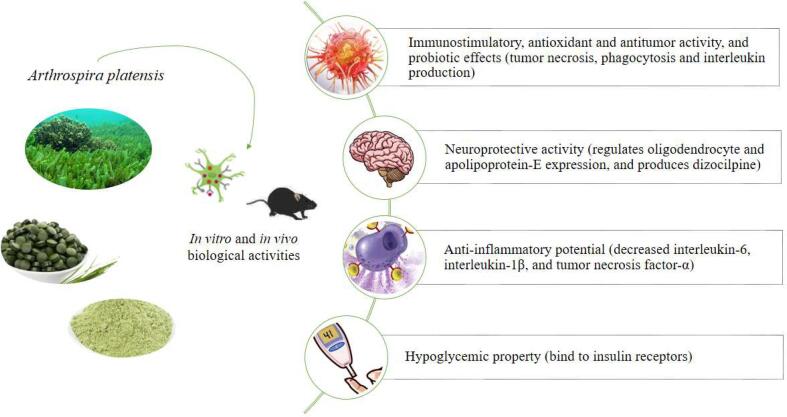
Biological effects of Spirulina of importance to the pharmaceutical, medical, chemical, cosmetic, and food industries.

As already mentioned, algae are rich in nutritional components (lipids, proteins, carbohydrates, pigments, vitamins, and minerals) (Table 1) that can be used as ingredients in food products such as cookies, sweets, snacks, pasta, and soft drinks, as well as dietary supplements via capsules, powder or tablets (Kusmayadi, Leong, Yen, Huang, & Chang, 2021). Besides to their versatility for food applications, these algae have numerous benefits to human health, including probiotic effects, antioxidant, antibacterial, antiviral, anticancer, anti-inflammatory, and antidiabetic activities (Table 3). These properties make algae important in the food industry as an additive for developing products in the medical, chemical, cosmetic, and pharmaceutical fields (Fig. 3).

**Table 3:** Biological effects of *Spirulina*.

*In vitro*	Species	Dose/duration	Mechanism of action	Citation
Probiotic activity	*Spirulina platensis*	NI	Oligosaccharides from *spirulina* promoted the abundance, diversity, and composition of gut microbiota, especially stimulating the growth of *Bacteroides*, *Escherichia-Shigella, Megamonas, Megasphaera, Blautia, Bifidobacterium* and *Lactobacillus*. In addition, by maintaining intestinal homeostasis, oligosaccharides promote the development of beneficial microbes, defend the microbiota against pathogens, and protect gastrointestinal function and immunoregulation.	Cai et al. (2022)
Immunostimulatory and antitumor activity	*Spirulina platensis*	16.25–50 μg/mL	Heteropolysaccharides from *spirulina* significantly inhibited the growth of A549 lung cancer cells, immune-enhancing activity on macrophages by promoting the proliferation and phagocytosis capacity of cells and stimulating the secretion of NO, IL-1β, and TNF-α without toxicity.	Cai et al. (2022b)
Pulmonary anticancer effect	*Spirulina platensis*	500 μg/mL per 24 h	*Spirulina* damages cancer cells affecting the cell cycle and forcing their apoptosis through biochemical changes.	Tajvidi et al. (2021)
Antithrombotic properties	*Spirulina maxima*	NI	*Spirulina* polysaccharide extracts, protein extracts (especially phycocyanobilin), and lipid extracts inhibit platelet-activating factor (PAF) and thrombin.	Koukouraki et al. (2020)

*In vivo*	Species	Dose/duration	Mechanism of action	Citation
Growth performance	*Spirulina platensis*	10 g/kg *Spirulina* + 0.1 mg/kg Se-SP	Diets fed with *Spirulina* and Se-SP significantly increased body weight and the production efficiency factor (313.50) of broilers.	Abdel-Moneim et al. (2022)
Antioxidant activity			The polyunsaturated fatty acids, phycocyanin, polyphenols, and β-Carotene present in *spirulina* can increase the antioxidant capacity of birds reared under thermal stress conditions.	
Improve humoral immunity			The dietary treatments of *Spirulina* and Se-SP contribute to alleviating the deleterious effect of thermal stress on humoral immunity by reducing the serum of immunoglobulin IgA, IgM, IgG, and antibodies Newcastle disease, avian influenza virus, and infectious bursal disease.	
Antimicrobial activity			*Spirulina* exhibited dose-dependent antimicrobial activities against ileal counts of total bacterial, total molds and yeast, coliform, *E. coli*, *Salmonella spp*., and *Enterococcus spp*. Thus, reducing the bacterial and fungal load of chickens.	
Dietary and ileal microbial potential			The bioactive compounds of *spirulina* have a probiotic effect capable of maintaining the homeostasis of the intestinal microbiota and controlling the colonization of pathogens in the chicken intestine.	
Hypoglycemic activity and bone protection	*Spirulina* spp.	300 mg/kg per 12 weeks	The reduction of the glucose level caused by chromium present in *spirulina* can prevent osteocytosis apoptosis and improve osteoblast differentiation. Thus, increasing the number of osteocytes and osteoblasts and protecting bones.	Ekeuku et al. (2021)
Wound healing potential	*Spirulina platensis*	NI	The topical supplementation with *Spirulina* demonstrated marked epithelization and complete connective tissue remodeling. These processes occur by improving the wound healing process by increasing angiogenesis and collagen deposition. Besides, the level of VEGF expression within the endothelial cells of the blood capillaries or fibroblastic cells was markedly expressed in *spirulina* treatment within the mature granulation tissue.	Elbialy et al. (2021)
Antioxidant activity	*Spirulina platensis*	500 and 1000 mg/kg per 30 days	The C-phycocyanin obtained from *spirulina* at 500 mg/kg, and 1000 mg/kg resulted in a significant enhancement of serum SOD activity higher than that of vitamin E.	Grover et al. (2021)
Immunomodulatory property			The C-phycocyanin suppresses the synthesis of pro-inflammatory cytokines, interferon-γ (IFN-γ), and TNF- α. In addition, the C-phycocyanin enhances the levels of anti-inflammatory cytokines, such as IL-10, in a concentration-dependent manner.	
Neuroprotective effects	*Spirulina platensis*	180 mg/kg	*Spirulina* regulates the hyperactive dopaminergic system by antioxidant effects. Consequently, it reduces the hyperactive motor deficits caused due to psychotic symptoms induced by dizocilpine.	Haider et al. (2021)
Cognitive enhancement	*Spirulina platensis*	1–2 % (w/w) per 16 weeks	*Spirulina* inhibited Aβ accumulation, tau-hyperphosphorylation, and neuroinflammation in the hippocampus.	Zhou et al. (2021)
Protection against oxidative damage	*Spirulina platensis*	400 mg/kg during the gestation and lactation period	After protein malnutrition, cellular changes in the hippocampus are partially restored after maternal *spirulina* protein supplementation. In addition, it reduced the astrocytes and activation of microglia, and increased cerebral cortical thickness, which is a better morphology of neuronal cells.	Sinha et al. (2020b)
Neuroprotective effects and cognitive enhancement	*Spirulina platensis*	400 mg/kg during the gestation and lactation period	*Spirulina* protein restores neurocognitive outcomes by reducing microglial activation, displacing the microglial phenotype to the neuroprotective profile, and promoting a positive increase in body and brain weight, maturation of vestibulocerebellar, tactile, and proprioceptive systems.	Sinha et al. (2020a)
Improves memory deficit	*Spirulina platensis*	2 mg/kg per 14 days	*Spirulina* improves scopolamine induced-memory deficit by inhibiting oxidative stress. This oxidative stress inhibition occurs by reducing malondialdehyde levels.	Ghanbari et al. (2019)
Hypolipidemic effect	*Spirulina maxima*	4.5 g/d per 45 days	*Spirulina* supplementation promoted linear reduction of total cholesterol, triglycerides, body fat, and body mass index.	Hernández-Lepe et al. (2019)

**Note:** NI – Not informed. Se: Selenium, NO: nitric oxide, IL-6: interleukin 6, IL-1β: interleukin-1β, TNF-α: tumor necrosis factor-α, MDA: the content of malondialdehyde, SOD: superoxide dismutase, GSH-Px: glutathione peroxidase, LPS: lipopolysaccharide, VEGF: vascular endothelial growth factor, SOD: Superoxide Dismutase.

### 5.1 Prebiotic effects

The prebiotic effect of *Spirulina platensis*, for example, results from its composition rich in oligosaccharides, which its consumption is capable of promoting the growth of intestinal microflora (Cai et al., 2022a). This probiotic activity is related to different structural characteristics, including molecular weight, the composition of the sugar residue, the type of bond between monosaccharides and stereochemistry, and the degree of polymerization of oligosaccharides in functional foods (Cai et al., 2022a)

### 5.2 Improvement of immune system and antitumor activity

In addition to probiotic effects, microalgae derivatives have the potential to improve the immune system and antitumor activity. The increase in the immune response, through the induction of tumor necrosis synthesis, phagocytosis, and the production of interleukin, results from the wide range of sulfate groups, sulfate esters, and amine residues present in *S. platensis* polysaccharides (Cai et al., 2022b). Concomitant, the antioxidant activity of *S. platensis* is also associated with activity against lung cancer cells. The microalgae extract affects the cell cycle of the A549 lung cancer cell line, increasing the production of reactive oxygen species and cell membrane lipid peroxidation. Its potential for cancer cell treatments lies mainly in the significant inhibitory effects on cancer cells while protecting normal cells (Tajvidi et al., 2021). Marková et al. (2020) also demonstrated inhibitory effects on the migration and invasion of pancreatic cancer (PA-TU-8902) provided by the extract of Spirulina. Furthermore, the extract allowed the regulation of mRNA and VEGF-A protein expressions in tumor cells and inhibiting ERK activation and suppressing the expression of ERK-regulated proteins. Thus, the anticancer effect of Spirulina was associated with suppressive effects on the migration and invasiveness of tumor cells with different antiangiogenic characteristics.

### 5.3 Neuroprotective effects

The inhibitory effects of inflammatory markers also contribute to neuroprotective activities, especially as a preventive and therapeutic measure against schizophrenia. The study by Haider et al. (2021) demonstrated that *S. platensis* administration could improve dizocilpine-induced behavioral deficits, regulate neurotransmission, restore immune response dysfunction by reducing inflammatory cytokines, and regulate dysfunction over oligodendrocyte and apolipoprotein-E expression. Its neuroprotective capacity may also be employed to improve scopolamine-induced memory deficit, protecting mice against neuronal damage (Ghanbari, Vafaei, Naghibi nasab, Attarmoghaddam, Bandegi, & Moradi- Kor, 2019). *Spirulina* spp. neuroprotective actions may also be evidenced in slowing the progression of multiple sclerosis (MS). In the study by Cervantes-Llanos et al. (2018), the oral (200 mg/kg) and daily intake of C-phycocyanin, a phycobiliprotein extracted from *S. platensis*, improves the quality of life of individuals with MS through the control of neuroinflammation through the modulation of redox processes and myelination/demyelination involved in this disease.

### 5.4 Anti-inflammatory properties

*Spirulina* spp. enriched with selenium improves its anti-inflammatory potential. The study by Jiang et al. (2022), for example, presents the effect of *S. platensis*-selenium (Se-SP) in the attenuation of inflammation induced by lipopolysaccharides. As a result, the anti-inflammatory effect of Se-SP is evidenced by the decrease in cytokine levels (74 % interleukin-6, 40.45 % interleukin-1β, and 42.28 % tumor necrosis factor-α). In addition, its antioxidant property protects cells against oxidative damage by decreasing nitric oxide (64.84 %) and malondialdehyde content (69.07 %) and increasing levels of superoxide dismutase and glutathione peroxidase enzymes. Se-SP is also used as growth promoters, antioxidants, immunostimulants, and antimicrobials (decreases pathogenic microorganisms in the feed and intestinal microbiota) in heat-stressed broilers (Abdel-Moneim et al., 2022).

Spirulina's anti-inflammatory potential also contributes to skin and burn healing. The study by Elbialy et al. (2021) demonstrated that topical use of *S. platensis* on wounds and burns is promising. Its application significantly improves the expression levels of vascular endothelial growth factor (VEGF) and fibrosis-related genes such as transforming growth factor-β (TGF-β), which play a fundamental role in skin repair. Furthermore, histopathological examination revealed cellular inflammatory infiltration (which leads to endothelial migration and production of chemotactic agents), angiogenesis (granulation tissue formation), epithelialization, extracellular matrix deposition (collagen hyperproliferation), and wound contraction (scar decrease). In addition, *Spirulina spp*. has antithrombotic properties. In the study by Koukouraki, Tsoupras, Sotiroudis, Demopoulos, and Sotiroudis (2020), *Spirulina maxima* lipid polysaccharide and protein extracts showed inhibitory effects against platelet aggregation induced by inflammatory and thrombotic mediators. Therefore, it is considered a potential food supplement and nutraceutical against inflammation, thrombosis, and related disorders.

### 5.5 Effects on metabolism disorders

*Spirulina spp*. is considered a superfood trend, which has been highlighted for its potential as a beneficial adjuvant therapy, especially in the treatment of diabetes and osteoporosis. The hypoglycemic property of *S. platensis* is associated with the presence of chromium, which, when binding to peptides that bind to insulin receptors, increases the activity of lowering plasma glucose levels (Ekeuku et al., 2021). In addition, hepatoprotective properties, protection of β cells against free radical damage, and the ability to increase vitamin D in the blood are correlated with these microalgae. These characteristics can improve bone formation, as evidenced in the study by Ekeuku et al. (2021), by increasing osteocalcin expression and the number of osteocytes/osteoblasts. Thus, its use associated with the drug metformin stimulates osteoblast proliferation and prevents osteocytosis apoptosis.

*S. platensis* supplementation, especially during pregnancy and lactation, has shown beneficial neuroprotective effects against the negative implications of malnutrition, reactive gliosis, and neurodegeneration (Sinha, Patro, Tiwari, & Patro, 2020b), thus regulating expression levels. Brain-derived neurotrophic factor, receptor tyrosine kinase, and receptor phosphotyrosine kinase. Furthermore, maternal supplementation with *S. platensis* protein can beneficially modulate neurobehavioral and cognitive deficits associated with malnutrition, promoting the offspring better cognition of neurological reflexes, greater neuromuscular strength, normalization of hyperactivity, and better spatial learning and memory (Sinha, Patro, & Patro, 2020a).

In dietary terms, *S. maxima* consumption increases the hypolipidemic effect in overweight and dyslipidemic men, reducing total cholesterol, triglycerides, body fat, and body mass index (Hernández-Lepe et al., 2019). Yet, it may improve the lipid profile of individuals with type 2 diabetes, metabolic syndrome, overweight, or obesity (Bohórquez-Medina et al., 2021).

In this context, the numerous biological properties of *Spirulina spp*. and its derivatives can go beyond its food and dietary supplement function and can be expanded in the medical field for various therapeutic functions, mainly against diseases associated with the accumulation of free radicals and oxidative stress, such as multiple sclerosis, Alzheimer's, Parkinson's and neurotoxic exposures (Cervantes-Llanos et al., 2018, Han et al., 2021, Sinha et al., 2020a).

## 6 Final considerations

The growing interest in healthy foods drives the development of research focused on discovering functional foods. In this sense, this review shows that Spirulina, a microalga with a rich nutritional and phytochemical composition, can be considered for the development of products with functional purposes. Among the wide range of Spirulina species, our bibliographic research showed that the most studied and applied species are limited to the species *S. platensis* and *S. maxima*, mainly due to their high productivity. Also, there are more publications involving the species *S. platensis.* Spirulina presents promising composition (carotenoids, phenolic compounds, phycocyanin, chlorophylls). It has been used as a nutritional supplement (powder, flakes, or capsules) and has been applied in food products of easy acceptance, such as cookies, pasta, sauces, ice cream, and snacks.

However, applications in food formulations represent a challenge, due to the taste, odor, which are limiting points, and color needs to be evaluated for an attractive sensory acceptance. In addition, new nutrient and phytochemical protection techniques are required to preserve Spirulina's important biological properties. Although the number of publications involving Spirulina is increasing year by year, further works are still needed to overcome the forementioned limitations, for example exploring microencapsulation as a solution and the biomass of Spirulina as much more than just a biomass, but a valuable ingredient for different formulations.

Studies addressed in this review show that biological activities such as immunostimulant, antioxidant, antitumor, neuroprotective, anti-inflammatory, hypoglycemic properties, and probiotic effects also make Spirulina a potential additive in the formulation of products in the medical, chemical, cosmetic, and pharmaceutical areas. Thus, the bibliographic synthesis of the effects of *Spirulina* spp. demonstrated that the microalga is an excellent ally to human health and can be incorporated into different food formulations, in its integral form or microencapsulated.

Based on the important biological activities attributed to Spirulina, future projections can be made regarding the application of this cyanobacterium. In addition to the biological characteristics, the nutritional properties of Spirulina make this ingredient promising for applications such as supplements, beverages, and foods with functional purposes. Bakery products such as cookies and bread can be functionalized with this microalga. Spirulina extract-based capsules and nutraceuticals may also be developed. This cyanobacterium can also be applied in the formulation of active packaging and dressings in biomedicine.

This review expands knowledge regarding new sources with functional purposes, such as the Spirulina microalgae. Studying the nutritional and phytochemical composition and biological effects allows the development of new scientific research, providing the use of this additive in different technological segments.

## Declaration of Competing Interest

The authors declare that they have no known competing financial interests or personal relationships that could have appeared to influence the work reported in this paper.

## References

[b0005] Abdel-Moneim A.M.E., Shehata A.M., Selim D.A., El-Saadony M.T., Mesalam N.M., Saleh A.A. (2022). Spirulina platensis and biosynthesized selenium nanoparticles improve performance, antioxidant status, humoral immunity and dietary and ileal microbial populations of heat-stressed broilers. Journal of Thermal Biology.

[b0010] Alagawany M., Taha A.E., Noreldin A., El-Tarabily K.A., Abd El-Hack M.E. (2021). Nutritional applications of species of Spirulina and Chlorella in farmed fish: A review. Aquaculture.

[b0015] Allied Market Research (2021). Spirulina Market by Type (Arthrospira Platensis and Arthrospira Maxima), Application (Nutraceuticals, Food & Beverages, Cosmetics, Animal Feed, and Others), and Formulation (Powder. *Tablet & Capsule, Liquid, and Granule & Gelling Agent): Global Opportunit*. Available online.

[b0020] Almeida L.M.R., da Cruz L.F., Machado B.A.S., Nunes I.L., Costa J.A.V., Ferreira E. de S., Souza C.O. de (2021). Effect of the addition of Spirulina sp. biomass on the development and characterization of functional food. Algal Research.

[b0025] Alongi M., Anese M. (2021). Re-thinking functional food development through a holistic approach. Journal of Functional Foods.

[b0030] Andrade B.B., Cardoso L.G., Assis D. de J., Costa J.A.V., Druzian J.I., Lima S.T. da C. (2019). Production and characterization of Spirulina sp. LEB 18 cultured in reused Zarrouk’s medium in a raceway-type bioreactor. Bioresource Technology.

[b0035] Bashir S., Sharif M.K., Butt M.S., Shahid M. (2016). Functional properties and amino acid profile of spirulina platensis protein isolates. Pakistan Journal of Scientific and Industrial Research Series B: Biological Sciences.

[b0040] Bigliardi B., Galati F. (2013). Innovation trends in the food industry: The case of functional foods. Trends in Food Science and Technology.

[b0045] Bohórquez-Medina S.L., Bohórquez-Medina A.L., Benites Zapata V.A., Ignacio-Cconchoy F.L., Toro-Huamanchumo C.J., Bendezu-Quispe G., Hernandez A.V. (2021). Impact of spirulina supplementation on obesity-related metabolic disorders: A systematic review and meta-analysis of randomized controlled trials. NFS Journal.

[b0060] Cai B., Yi X., Han Q., Pan J., Chen H., Sun H., Wan P. (2022). Structural characterization of oligosaccharide from Spirulina platensis and its effect on the faecal microbiota in vitro. Food Science and Human Wellness.

[b0065] Cai B., Zhao X., Luo L., Wan P., Chen H., Pan J. (2022). Structural characterization, and in vitro immunostimulatory and antitumor activity of an acid polysaccharide from Spirulina platensis. International Journal of Biological Macromolecules.

[b0070] Carpentieri S., Larrea-Wachtendorff D., Donsì F., Ferrari G. (2022). Functionalizationof pasta through the incorporation of bioactive compounds from agri-food by-products: Fundamentals, opportunities, and drawbacks. Trends in Food Science & Technology.

[b0075] Carvalho L.F., Moreira J.B., Oliveira M.S., Costa J.A.V. (2017). Novel food supplements formulated with Spirulina to meet athletes’ needs. Brazilian Archives of Biology and Technology.

[b0080] Cervantes-Llanos M., Lagumersindez-Denis N., Marín-Prida J., Pavón-Fuentes N., Falcon-Cama V., Piniella-Matamoros B., Pentón-Rol G. (2018). Beneficial effects of oral administration of C-Phycocyanin and Phycocyanobilin in rodent models of experimental autoimmune encephalomyelitis. Life Sciences.

[b0315] da Silva S.P., Valle A.F., Perrone D. (2021). Microencapsulated Spirulina maxima biomass as an ingredient for the production of nutritionally enriched and sensorially well-accepted vegan biscuits. LWT.

[b0090] da Silva P.C., Toledo T., Brião V., Bertolin T.E., Costa J.A.V. (2021). Development of extruded snacks enriched by bioactive peptides from microalga Spirulina sp. LEB 18. *Food*. Bioscience.

[b0095] de Amarante M.C.A., Braga A.R.C., Sala L., Kalil S.J. (2020). Colour stability and antioxidant activity of C-phycocyanin-added ice creams after in vitro digestion. Food Research International.

[b0055] Bortolini D.G., Maciel G.M., de Fernandes I., Rossetto R., Brugnari T., Ribeiro T., Ribeiro V.R., Haminiuk C.W.I. (2022). Biological potential and technological applications of red fruits: An overview. Food Chemistry Advances.

[b0050] Bortolini, D. G., Haminiuk, C. W. I., Pedro, A. C., Fernandes, I. de A. A., & Maciel, G. M. (2021). Processing, chemical signature and food industry applications of Camellia sinensis teas: An overview. *Food Chemistry: X*, *12*, 100160. doi: 10.1016/j.fochx.2021.100160. 10.1016/j.fochx.2021.100160 PMC8605308 34825170

[b0100] de Medeiros, V. P. B., da Costa, W. K. A., da Silva, R. T., Pimentel, T. C., & Magnani, M. (2021). Microalgae as source of functional ingredients in new-generation foods: challenges, technological effects, biological activity, and regulatory issues. doi: 10.1080/10408398.2021.1879729. 10.1080/10408398.2021.1879729 33544001

[b0105] de Morais E.G., Druzian J.I., Nunes I.L., de Morais M.G., Costa J.A.V. (2019). Glycerol increases growth, protein production and alters the fatty acids profile of Spirulina (Arthrospira) sp LEB 18. Process Biochemistry.

[b0110] de Oliveira, T. T. B., dos Reis, I. M., de Souza, M. B., Bispo, E. da S., Maciel, L. F., Druzian, J. I., Tavares, P. P. L. G., Cerqueira, A. de O., Morte, E. dos S. B., Glória, M. B. A., Deus, V. L., & de Santana, L. R. R. (2021). Microencapsulation of Spirulina sp. LEB-18 and its incorporation in chocolate milk: Properties and functional potential. *LWT*, *148*, 111674. doi: 10.1016/J.LWT.2021.111674.

[b0115] Ekeuku S.O., Chong P.N., Chan H.K., Mohamed N., Froemming G.R.A., Okechukwu P.N. (2021). Spirulina supplementation improves bone structural strength and stiffness in streptozocin-induced diabetic rats. Journal of Traditional and Complementary Medicine.

[b0120] El-baky H.H.A., El Baz F.K., El-baroty G.S. (2009). Production of phenolic compounds from Spirulina maxima microalgae.pdf. African Journal of Biotechnology.

[b0125] Elbialy Z.I., Assar D.H., Abdelnaby A., Asa S.A., Abdelhiee E.Y., Ibrahim S.S., Atiba A. (2021). Healing potential of Spirulina platensis for skin wounds by modulating bFGF, VEGF, TGF-ß1 and α-SMA genes expression targeting angiogenesis and scar tissue formation in the rat model. Biomedicine and Pharmacotherapy.

[b0135] Freitas B.C.B., Santos T.D., Moreira J.B., Zanfonato K., Morais M.G., Costa J.A.V. (2019). Novel foods: A meal replacement shake and a high-calorie food supplemented with Spirulina biomass. International Food Research Journal.

[b0140] Ghanbari A., Vafaei A.A., Naghibi nasab F.S., Attarmoghaddam M., Bandegi A.R., Moradi-Kor N. (2019). Spirulina microalgae improves memory deficit induced by scopolamine in male pup rats: Role of oxidative stress. South African Journal of Botany.

[b0145] Bortolini D.G., Barros L., Maciel G.M., Brugnari T., Modkovski T.A., Fachi M.M., Pontarolo R., Pinela J., Ferreira I.C.F.R., Haminiuk C.W.I. (2022). Bioactive profile of edible nasturtium and rose flowers during simulated gastrointestinal digestion. Food Chemistry.

[b0130] Faresin, L. da S., Devos, R. J. B., Reinehr, C. O., & Colla, L. M. (2022). Development of ice cream with reduction of sugar and fat by the addition of inulin, Spirulina platensis or phycocyanin. *International Journal of Gastronomy and Food Science*, *27*, 100445. doi: 10.1016/j.ijgfs.2021.100445.

[b0150] Granato D., Barba F.J., Bursać Kovačević D., Lorenzo J.M., Cruz A.G., Putnik P. (2020). Functional Foods: Product Development, Technological Trends, Efficacy Testing. and Safety.

[b0155] Grand View Research (2019a). Functional Foods Market Size, Share & Trends Analysis Report By Ingredient (Carotenoids, Prebiotics & Probiotics, Fatty Acids, Dietary Fibers), By Product, By Application, And Segment Forecasts. Available online: https://www.grandviewresearch.com/industry-analysis/functional-food-market.

[b0160] Grand View Research (2019b). *Functional Foods Market Worth $275.7 Billion By 2025 | CAGR: 7.9%*. Available online: https://www.grandviewresearch.com/press-release/global-functional-foods-market.

[b0165] Grochowicz J., Fabisiak A., Ekielski A. (2022). Importance of physical and functional properties of foods targeted to seniors. Journal of Future Foods.

[b0170] Grover P., Bhatnagar A., Kumari N., Narayan Bhatt A., Kumar Nishad D., Purkayastha J. (2021). C-Phycocyanin-a novel protein from Spirulina platensis- In vivo toxicity, antioxidant and immunomodulatory studies. Saudi Journal of Biological Sciences.

[b0175] Gur J., Mawuntu M., Martirosyan D. (2018). FFC’s advancement of functional food definition. Functional Foods in Health and Disease.

[b0180] Haider S., Shahzad S., Batool Z., Sadir S., Liaquat L., Tabassum S., Perveen T. (2021). Spirulina platensis reduces the schizophrenic-like symptoms in rat model by restoring altered APO-E and RTN-4 protein expression in prefrontal cortex. Life Sciences.

[b0185] Han P., Li J., Zhong H., Xie J., Zhang P., Lu Q., Zhou W. (2021). Anti-oxidation properties and therapeutic potentials of spirulina. Algal Research.

[b0190] Harrison L., Smith R. (2016). Developing food products for consumers concerned with physical activity, sports, and fitness. Developing Food Products for Consumers with Specific Dietary Needs.

[b0195] Hernández-Lepe M.A., Wall-Medrano A., López-Díaz J.A., Juárez-Oropeza M.A., Luqueño-Bocardo O.I., Hernández-Torres R.P., Ramos-Jiménez A. (2019). Hypolipidemic Effect of Arthrospira (Spirulina) maxima Supplementation and a Systematic Physical Exercise Program in Overweight and Obese Men: A Double-Blind, Randomized, and Crossover Controlled Trial. Marine Drugs.

[b0200] Hynstova V., Sterbova D., Klejdus B., Hedbavny J., Huska D., Adam V. (2018). Separation, identification and quantification of carotenoids and chlorophylls in dietary supplements containing Chlorella vulgaris and Spirulina platensis using High Performance Thin Layer Chromatography. Journal of Pharmaceutical and Biomedical Analysis.

[b0205] Iamtham S., Sornchai P. (2022). Biofixation of CO2 from a power plant through large-scale cultivation of Spirulina maxima. South African Journal of Botany.

[b0210] Industry ARC (2021). *Spirulina Powder Market - Forecast (2022 - 2027)*. Available online: https://www.industryarc.com/Report/19629/spirulina-powder-market.html?https://www.industryarc.com/Report/19629/spirulina-powder-market.html&gclid=Cj0KCQjwxveXBhDDARIsAI0Q0x2bzRXcN9yjaMawMhblXMaLDaKutFCeKcBfKxis8c3ToLJAyDfeaRMaAii-EALw_wcB# (acessed on 17 August 2022).

[b0215] Jiang P., Meng J., Zhang L., Huang L., Wei L., Bai Y., Li S. (2022). Purification and anti-inflammatory effect of selenium-containing protein fraction from selenium-enriched Spirulina platensis. Food Bioscience.

[b0220] Koukouraki P., Tsoupras A., Sotiroudis G., Demopoulos C.A., Sotiroudis T.G. (2020). Antithrombotic properties of Spirulina extracts against platelet-activating factor and thrombin. Food Bioscience.

[b0225] Kusmayadi A., Leong Y.K., Yen H.W., Huang C.Y., Chang J.S. (2021). Microalgae as sustainable food and feed sources for animals and humans – Biotechnological and environmental aspects. Chemosphere.

[b0230] Lafarga T., Fernández-Sevilla J.M., González-López C., Acién-Fernández F.G. (2020). Spirulina for the food and functional food industries. Food Research International.

[b0235] Lafarga T., Sánchez-Zurano A., Villaró S., Morillas-España A., Acién G. (2021). Industrial production of spirulina as a protein source for bioactive peptide generation. Trends in Food Science and Technology.

[b0240] Li T.-T., Tong A.-J., Liu Y.-Y., Huang Z.-R., Wan X.-Z., Pan Y.-Y., Zhao C. (2019). Polyunsaturated fatty acids from microalgae Spirulina platensis modulates lipid metabolism disorders and gut microbiota in high-fat diet rats. Food and Chemical Toxicology.

[b0245] Lim S.J., Chang L.S., Fazry S., Wan Mustapha W.A., Babji A.S. (2021). Functional food & ingredients from seaweed, edible bird’s nest and tropical fruits: A translational research. LWT.

[b0250] Los P.R., Simões D.R.S., de Leone R., S., Bolanho, B. C., Cardoso, T., & Danesi, E. D. G. (2018). Viability of peach palm by-product, Spirulina platensis, and spinach for the enrichment of dehydrated soup. Pesquisa Agropecuária Brasileira.

[b0255] Lucas B.F., de Morais M.G., Santos T.D., Costa J.A.V. (2017). Effect of spirulina addition on the physicochemical and structural properties of extruded snacks. Food Science and Technology.

[b0260] Lucas B.F., de Morais M.G., Santos T.D., Costa J.A.V. (2018). Spirulina for snack enrichment: Nutritional, physical and sensory evaluations. LWT.

[b0265] Lucas B.F., da Rosa, de Carvalho, de Morais, Costa J.A. V. (2020). Snack bars enriched with spirulina for schoolchildren nutrition. Food Science and Technology.

[b0270] Machu L., Misurcova L., Ambrozova J.V., Orsavova J., Mlcek J., Sochor J., Jurikova T. (2015). Phenolic content and antioxidant capacity in algal food products. Molecules.

[b0275] Marková I., Koníčková R., Vaňková K., Leníček M., Kolář M., Strnad H., Vítek L. (2020). Anti-angiogenic effects of the blue-green alga Arthrospira platensis on pancreatic cancer. Journal of Cellular and Molecular Medicine.

[b0280] Martins M., Albuquerque C.M., Pereira C.F., Coutinho J.A.P., Neves M.G.P.M.S., Pinto D.C.G.A., Ventura S.P.M. (2021). Recovery of Chlorophyll a Derivative from Spirulina maxima: Its Purification and Photosensitizing Potential. ACS Sustainable Chemistry and Engineering.

[b0285] Masuda K., Chitundu M. (2019). Multiple micronutrient supplementation using spirulina platensis and infant growth, morbidity, and motor development: Evidence from a randomized trial in Zambia. PLoS ONE.

[b0290] Menegotto A.L.L., de Souza L.E.S., Colla L.M., Costa J.A.V., Sehn E., Bittencourt P.R.S., M., Canan, C., & Colla, E. (2019). Investigation of techno-functional and physicochemical properties of Spirulina platensis protein concentrate for food enrichment. LWT.

[b0295] Meticulous Research (2021). *Spirulina Market by Distribution Channel (Consumer Channel, Business Channel), Product Type (Powder, Tablets, Capsules, Flakes, Phycocyanin Extract), Application (Nutraceuticals, Food and Beverages, Agriculture, Animal Feed) - Global Forecast to 2028*. Available online: https://www.meticulousresearch.com/product/spirulina-market-5070 (acessed on 17 August 2022).

[b0300] Michael A., Kyewalyanga M.S., Lugomela C.V. (2019). Biomass and nutritive value of Spirulina (Arthrospira fusiformis) cultivated in a cost-effective medium. Annals of Microbiology.

[b0305] Neves M.I.L., Silva E.K., Meireles M.A.A. (2021). Natural blue food colorants: Consumer acceptance, current alternatives, trends, challenges, and future strategies. Trends in Food Science & Technology.

[b0310] Park W.S., Kim H.J., Li M., Lim D.H., Kim J., Kwak S.S., Ahn M.J. (2018). Two classes of pigments, carotenoids and c-phycocyanin, in spirulina powder and their antioxidant activities. Molecules.

[b0320] Rodrigues R.D.P., de Castro F.C., de Santiago-Aguiar R.S., Rocha M.V.P. (2018). Ultrasound-assisted extraction of phycobiliproteins from Spirulina (Arthrospira) platensis using protic ionic liquids as solvent. Algal Research.

[b0325] Rodrigues E.F., Vendruscolo L.P., Bonfante K., Reinehr C.O., Colla E., Colla L.M. (2020). Phycocyanin as substitute for texture ingredients in ice creams. British Food Journal.

[b0330] Rossetto R., Maciel G.M., Bortolini D.G., Ribeiro V.R., Haminiuk C.W.I. (2020). Acai pulp and seeds as emerging sources of phenolic compounds for enrichment of residual yeasts (Saccharomyces cerevisiae) through biosorption process. LWT - Food Science and Technology.

[b0335] Silva S.C., Ferreira I.C.F.R., Dias M.M., Barreiro M.F. (2020). Microalgae-Derived Pigments: A 10-Year Bibliometric Review and Industry and Market Trend Analysis. Molecules.

[b0340] Sinha S., Patro N., Patro I.K. (2020). Amelioration of neurobehavioral and cognitive abilities of F1 progeny following dietary supplementation with Spirulina to protein malnourished mothers. Brain, Behavior, and Immunity.

[b0345] Sinha S., Patro N., Tiwari P.K., Patro I.K. (2020). Maternal Spirulina supplementation during pregnancy and lactation partially prevents oxidative stress, glial activation and neuronal damage in protein malnourished F1 progeny. Neurochemistry International.

[b0350] Stafussa A.P., Maciel G.M., Bortolini D.G., Maroldi W.V., Ribeiro V.R., Fachi M.M., Haminiuk C.W.I. (2021). Bioactivity and bioaccessibility of phenolic compounds from Brazilian fruit purees. Future Foods.

[b0355] Tajvidi E., Nahavandizadeh N., Pournaderi M., Pourrashid A.Z., Bossaghzadeh F., Khoshnood Z. (2021). Study the antioxidant effects of blue-green algae Spirulina extract on ROS and MDA production in human lung cancer cells. Biochemistry and Biophysics Reports.

[b0360] Tavanandi H.A., Raghavarao K.S.M.S. (2020). Ultrasound-assisted enzymatic extraction of natural food colorant C-Phycocyanin from dry biomass of Arthrospira platensis. LWT - Food Science and Technology.

[b0365] Teixeira V.M.C., da Silva R.F.G., Gonçalves O.H., Pereira C., Barros L., Ferreira I.C.F.R., Leimann F.V. (2022). Chemometric approaches to evaluate the substitution of synthetic food dyes by natural compounds: The case of nanoencapsulated curcumin, spirulina, and hibiscus extracts. LWT.

[b0370] Thevarajah B., Nishshanka G.K.S.H., Premaratne M., Nimarshana P.H.V., Nagarajan D., Chan J.-S., Ariyadasa T.U. (2022). Large-scale production of Spirulina -based proteins and c-phycocyanin : A biorefinery approach. Biochemical Engineering Journal.

[b0375] USDA. (2022). Food Data Central. https://fdc.nal.usda.gov/fdc-app.html#/food-details/170091/nutrients (Accessed on 2 April 2022).

[b0380] Zen C.K., Tiepo C.B.V., da Silva R.V., Reinehr C.O., Gutkoski L.C., Oro T., Colla L.M. (2020). Development of functional pasta with microencapsulated Spirulina: Technological and sensorial effects. Journal of the Science of Food and Agriculture.

[b0385] Zhou T., Liu Y., Wang Q., Dou Q., Li X., Pan Y., Xue T. (2021). Spirulina platensis alleviates high fat diet-induced cognitive impairment in mice via the gut-brain axis. Journal of Functional Foods.

[b0390] Zhu B., Shen H., Li Y., Liu Q., Jin G., Han J., Pan K. (2020). Large-Scale Cultivation of Spirulina for Biological CO2 Mitigation in Open Raceway Ponds Using Purified CO2 From a Coal Chemical Flue Gas. Frontiers in Bioengineering and Biotechnology.

